# Osteitis Fibrosa Cystica: The Hideous Face of Hyperparathyroidism

**DOI:** 10.7759/cureus.80271

**Published:** 2025-03-08

**Authors:** Ahmed Amine Jaouahar, Mehdi Chakib, Omar Maoujoud, Mohammed Asserraji, Nadir Zemraoui

**Affiliations:** 1 Nephrology and Hemodialysis, Avicenna Military Hospital, Marrakesh, MAR

**Keywords:** brown tumors, end-stage renal disease, hemodialysis, hyperparathyroidism, osteitis fibrosa cystica

## Abstract

Osteitis fibrosa cystica (OFC), the skeletal scourge of advanced hyperparathyroidism, unveils itself as a relentless destroyer of bone architecture, leaving behind a landscape of fragility and deformity. In the shadow of chronic kidney disease, where mineral imbalances reign, this rare but devastating entity emerges as a silent tormentor. We present the case of a hemodialysis patient who fell victim to this sinister complication, manifesting with excruciating bone pain, pathological fractures, and radiographic and histological evidence of cystic osteolytic lesions. Biological assessments revealed strikingly elevated parathormone levels, a hallmark of severely unbalanced parathyroid overactivity, fueling the relentless skeletal destruction. A technetium (99mTc) Sestamibi parathyroid scan was performed, showing a parathyroid adenoma and thus confirming tertiary hyperparathyroidism as the underlying cause. This case underscores the urgent need for early recognition, a vigilant therapeutic strategy, and potential parathyroidectomy to halt the merciless grip of OFC, a true testament to the havoc that dysregulated calcium-phosphorus metabolism can wreak on the human frame.

## Introduction

Secondary hyperparathyroidism (sHPT) is a major and common complication of chronic kidney disease (CKD). It is principally due to a deficiency in 1,25-dihydroxy-vitamin D3, leading to major disturbances in phosphocalcic metabolism [1]. Osteitis fibrosa cystica (OFC) is a rare but serious bone disorder resulting from excessive parathormone (PTH) activity, leading to increased bone resorption and fibrotic replacement of the marrow. Brown tumors represent a rare and severe manifestation of OFC [2]. The incidence of brown tumors in hemodialysis patients has significantly declined due to routine biological monitoring, which allows early diagnosis of sHPT before tumor development, and the advent of novel therapeutic options, including non-calcium-based phosphate binders, vitamin D analogs, and calcimimetics [1]. Unfortunately, the limited availability of these therapeutic agents in certain regions continues to result in the occurrence of brown tumors in hemodialysis patients. In patients with CKD, prolonged sHPT may evolve into tertiary hyperparathyroidism, characterized by autonomous parathyroid hyperplasia and persistently elevated PTH levels. This condition predisposes patients to severe bone pathology, including fractures, bone pain, and deformities [3]. The following case illustrates the monstrous clinical presentation of OFC in a hemodialysis patient and underscores the importance of early diagnosis and appropriate management strategies to mitigate the devastating skeletal consequences and improve patient's quality of life.

## Case presentation

**Figure 1:**
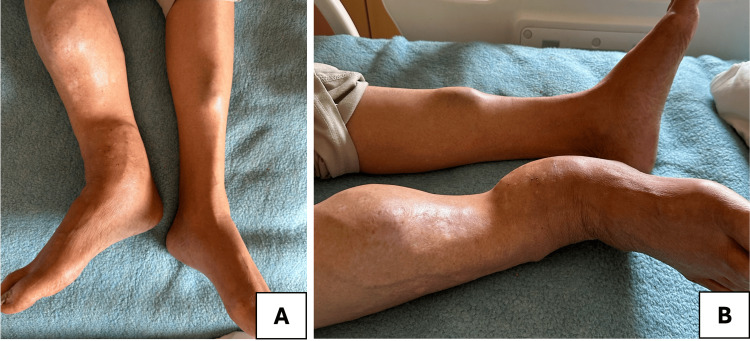
Deformities and swelling of the lower limb A: Frontal view; B: Lateral view

A 68-year-old man, on maintenance hemodialysis for 12 years due to end-stage renal disease secondary to diabetic nephropathy and a history of a suspicious pathological hip fracture operated six months ago, presented with progressive bone pain and fatigue. Physical examination revealed significant deformities and swelling of the lower limb, particularly the right tibia and fibula, with an abnormal bony prominence. The onset of symptoms was insidious, gradually developing over approximately four to five months prior to presentation. The most striking feature was the irregular and enlarged mass-like deformity involving the mid to distal portion of the right leg. The skin appears intact, with no visible ulcerations or signs of active infection (Figure 1).

Laboratory findings showed markedly elevated PTH levels, hypercalcemia, and normal phosphate levels, while inflammatory markers were negative (Table 1).

**Table 1:** Laboratory findings of the patient

Laboratory parameters	Patient values	Normal range
White blood cells	7.1 x 10^9^/L	4.0-10 x 10^9^/L
Hemoglobin	11.3 g/dL	12-16 g/dL
Platelets	310 x 10^9^/L	150-400 x 10^9^/L
C-reactive protein	2 mg/dL	<0.3 mg/dL
Serum calcium	118 mg/L	85-105 mg/L
Serum phosphate	42 mg/L	25-45 mg/L
Parathormone (PTH)	1980 pg/mL	15-65 pg/mL

**Figure 2:**
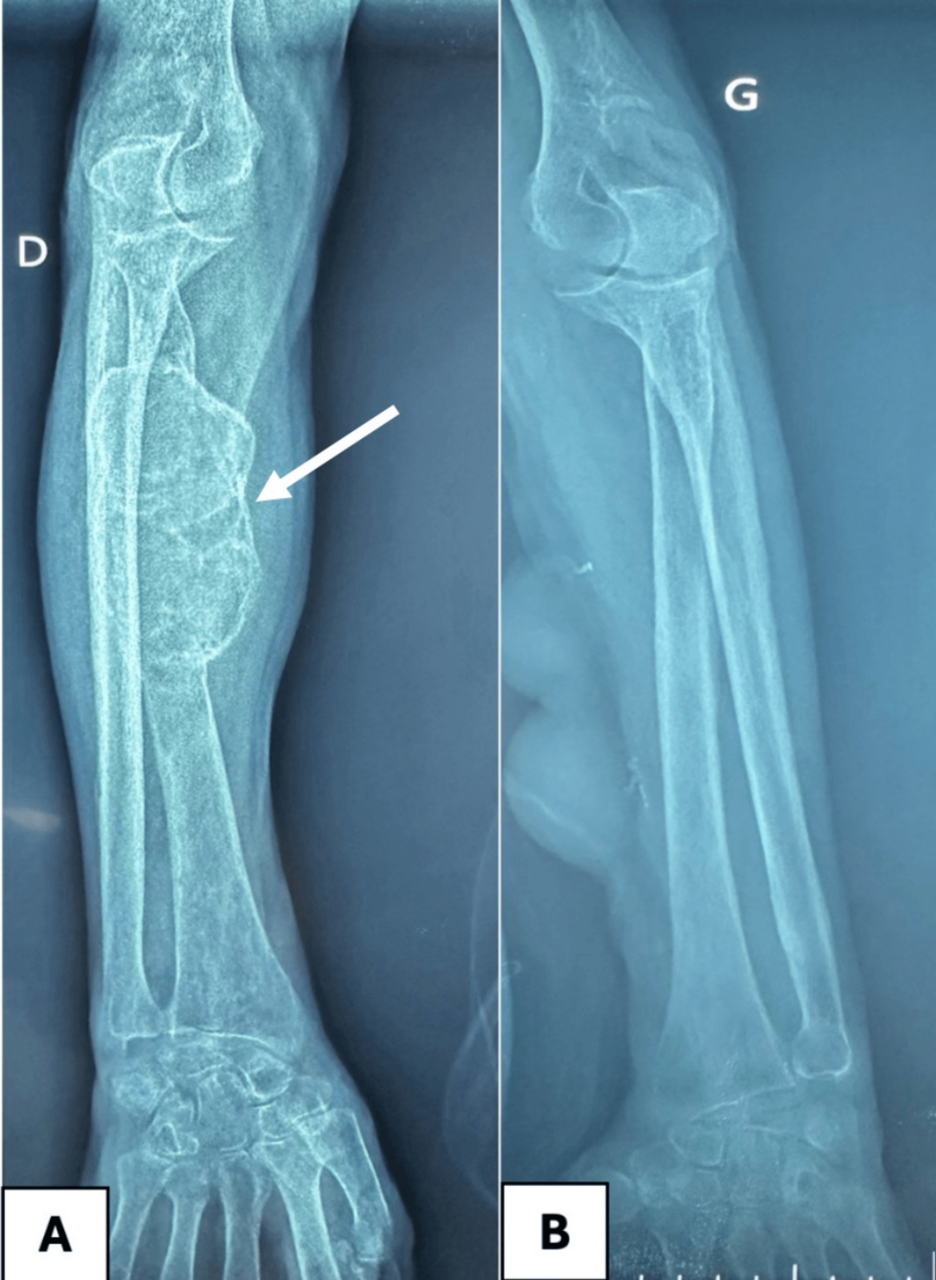
X-rays of both forearms Brown tumor aspect in the mid portion of the radius of the right forearm A: Right forearm; B: Left forearm

**Figure 3:**
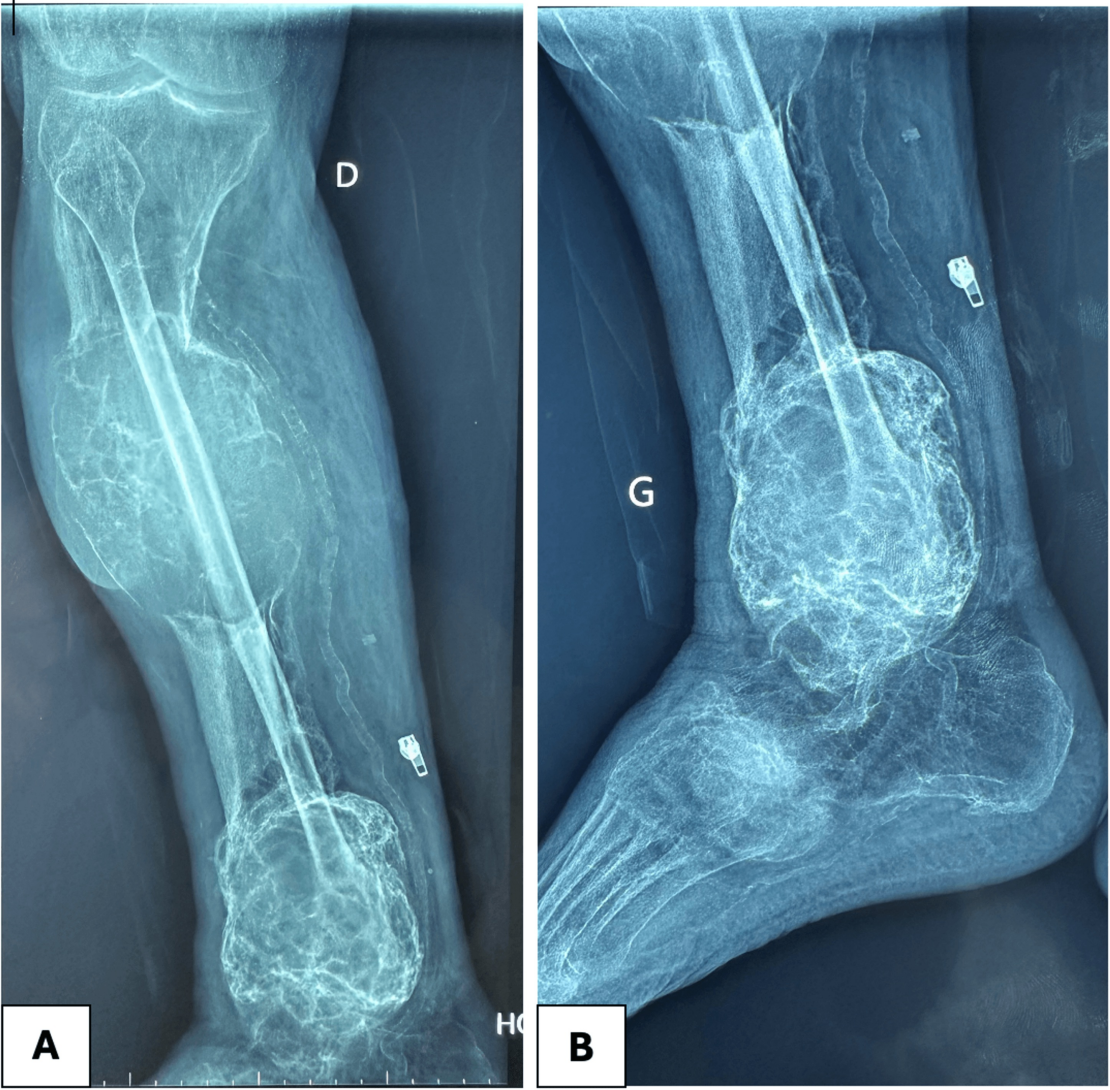
X-rays of the right lower limb lesions X-ray images (A and B) reveal large, well-defined, expansile lytic lesions affecting the distal and mid tibia. Cortical bone appears attenuated and partially disrupted, suggesting aggressive osteoclastic activity. The lesion has multiple internal septations, giving it a trabeculated or "cystic" appearance, characteristic of brown tumors. A: Frontal view; B: Lateral view

Radiographic imaging revealed subclinical lesions affecting the mid portion of the radius of the right upper limb. X-ray images of the lower limb showed multiple lytic bone lesions suggestive of brown tumors, subperiosteal resorption, and diffuse osteopenia (Figures 2, 3).

**Figure 4:**
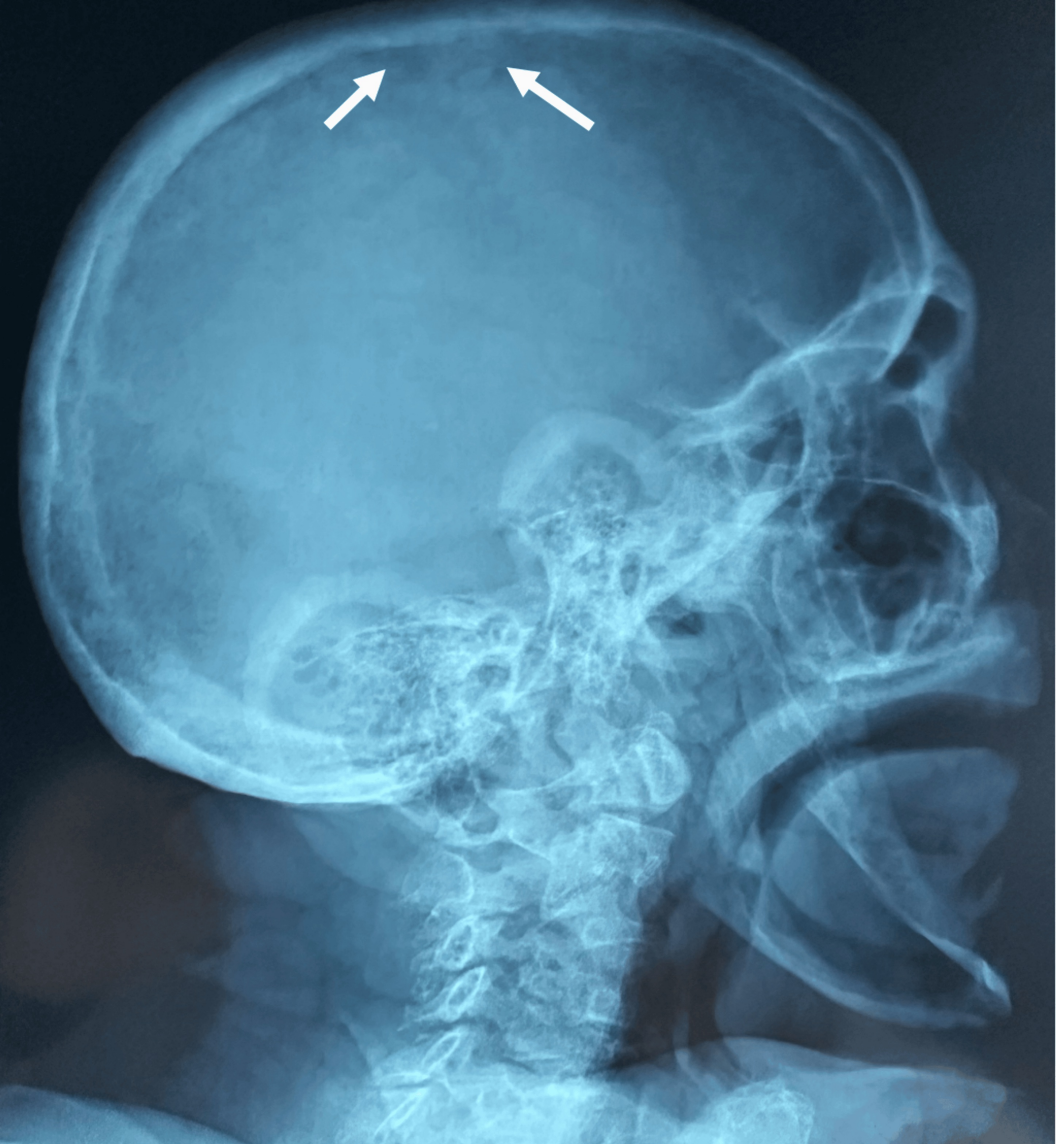
Skull X-ray showing "salt-and-pepper" sign

**Figure 5:**
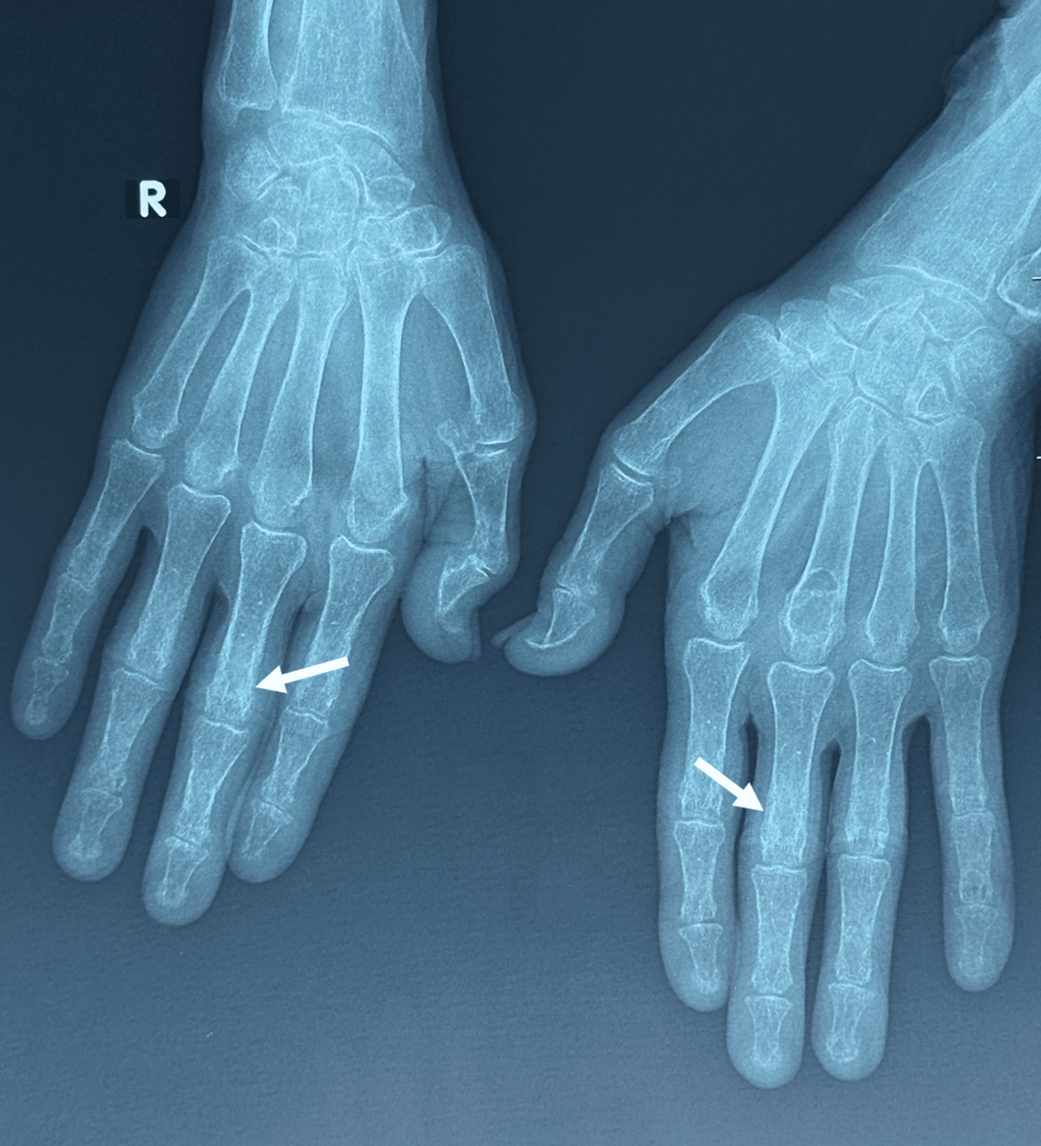
Hands X-ray showing subperiosteal resorption on both sides of some phalanges

In addition, a salt-and-pepper skull aspect was found along with subperiosteal resorption on both sides of some phalanges (Figures 4, 5).

**Figure 6:**
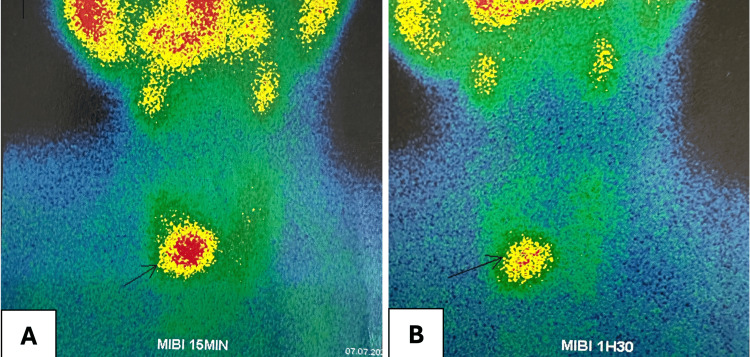
Tc-99m Sestamibi parathyroid scintigraphy showing intense uptake in the inferior right parathyroid gland A: Cervical images obtained 15 minutes after injection; B: Second acquisition 1.5 hours later

A Sestamibi parathyroid scan was performed, demonstrating intense uptake in the inferior right parathyroid gland, indicative of hyperplastic and autonomously functioning parathyroid tissue, confirming tertiary hyperparathyroidism (Figure 6).

The biopsy of the lesions was deemed unnecessary due to the highly suggestive clinical and biological context: the patient presents with CKD undergoing hemodialysis and a history of sHPT, likely progressing to tertiary hyperparathyroidism, accompanied by the associated multiple manifestations.

Given the severity of skeletal involvement and failure of medical therapy, the patient underwent subtotal parathyroidectomy with a PTH level dropping to 112 pg/mL immediately after surgery. There was a significant improvement in functional signs with disappearance of bone pain. Other clinical, biological, and imaging follow-ups are still ongoing.

## Discussion

First described in 1891 by Von Reckling Hausen, OFC is a fibro-cystic bone lesion resulting from excessive osteoclastic activity secondary to a state of hyperparathyroidism [4]. Brown tumors are the primary manifestation of fibrocystic osteitis; it is a characteristic skeletal complication that often mimics many neoplastic lesions [5]. This represents an uncommon complication, making up approximately 10% of all skeletal lesions. Its incidence is reported at 3% in primary hyperparathyroidism and ranges between 1.5% and 1.7% in sHPT among young adults. In over 80% of cases, it is attributed to the presence of a parathyroid adenoma [6]. Brown tumors predominantly affect young patients and women, exhibiting varying degrees of aggressiveness and risk of recurrence [7].

The clinical presentation is primarily characterized by bone pain, palpable masses, local swelling, and in some cases, pathological microfractures [7]. The predominant tumor locations include the ribs, sternum, clavicle, pelvis, and the diaphyseal regions of long bones. Spinal involvement is rarer and may occur with or without associated neurological symptoms [8]. 

The primary pathogenic mechanism leading to sHPT is hyperphosphatemia and a deficiency in 1,25-dihydroxycholecalciferol, which results in hypocalcemia. This hypocalcemia, in turn, stimulates an increased production and secretion of PTH by the parathyroid glands. PTH then acts on bone tissue, mobilizing calcium and phosphate and attempting to restore calcium levels to a near-normal range [7,8]. 

Hyperparathyroidism is characterized by high PTH levels, with variable calcium values (usually hypercalcemia or normocalcemia), hypophosphatemia, elevated total alkaline phosphatase levels (>240 IU/L), and increased bone-specific alkaline phosphatase (>25 ng/mL). Elevated PTH and alkaline phosphatase levels indicate active bone remodeling; however, there is no direct correlation between plasma PTH concentrations and the nature of the bone lesion, particularly in the case of brown tumors. Similarly, no significant correlation exists between the plasma levels of calcium and phosphorus and the presence of these skeletal lesions [9,10].

The positive diagnosis, essentially clinical and biological, should be supported by radiological investigations. In the early stages, bone lesions present as an intracortical resorption zone, which then evolves into a lytic bone lesion of variable size, either single or multiple. These lesions show intra-lesional trabeculations, which may appear blown or disrupt the cortical bone, thinning it, with peri-lesional condensation and, at times, polycyclic contours [11].

CT scanning is not systematically indicated and is primarily useful in cases of spinal or cranial involvement. The characteristic appearance of the tumor is that of expansive medullary lytic bone lesions, some with sclerotic margins, others with cortical destruction or associated soft-tissue masses [12]. MRI is particularly useful for vertebral localizations. The brown tumor appears as a lesion that is hypointense on T1-weighted images and hyperintense on T2-weighted images [13].

FDG PET-CT imaging of a brown tumor typically reveals significant hypermetabolic activity. This heightened metabolism is likely attributable to the presence of multinucleated giant cells and the high rate of intracellular metabolic processes occurring in macrophages within the lesions [14]. Sestamibi scintigraphy is recognized as the first-line diagnostic modality for detecting parathyroid adenomas, corroborating the hypothesis of a hyperparathyroidism-related brown tumor [15].

Histological examination of the tumor through biopsy is rarely performed but has been reported mainly in cases involving the mandible and spine or when the diagnosis is uncertain. Histological analysis confirms the diagnosis by revealing a zone of intense osteoclastic resorption with hypervascular inflammatory connective tissue, multinucleated giant cells, hemosiderin deposits, and areas of osteoid tissue formation [16]. It is important to emphasize that brown tumors are non-neoplastic lesions with no malignant potential, in contrast to true giant cell tumors, which carry a risk of malignant transformation and pulmonary metastases. Consequently, true giant cell tumors require radical surgical treatment [17].

The differential diagnosis includes multiple myeloma, osteosarcoma, bone metastasis from an osteophilic cancer, osteomyelitis, and Paget's disease. The elevated serum PTH levels and parathyroid uptake of 99mTc-Sestamibi strongly guide the diagnosis towards a brown tumor [18].

The treatment of brown tumors primarily focuses on correcting the underlying hyperparathyroidism. In cases where the condition is caused by a parathyroid adenoma, as seen in this patient, surgical removal of the adenoma is essential. Postoperatively, long-term calcium supplementation at high doses is usually required for at least 12 months. The prognosis is generally favorable once hyperparathyroidism is controlled, with regression of brown tumors typically occurring within a year after parathyroidectomy [19].

Brown tumors generally undergo spontaneous clinical and radiological regression following the correction of hyperparathyroidism. However, surgical intervention, including enucleation, curettage, or complete resection with reconstruction, may be necessary when functional prognosis is at risk. The surgical indication depends on the tumor’s progression after hyperparathyroidism treatment. Surgery is warranted in cases of abnormal tumor growth, excessively slow regression, or large, symptomatic lesions leading to functional impairment [20].

## Conclusions

OFC, particularly in the context of tertiary hyperparathyroidism, represents a devastating skeletal pathology in patients with CKD. The progressive and silent bone destruction highlights the insidious nature of this condition. Without prompt intervention, the relentless bone resorption can lead to irreversible deformities and fractures. Parathyroidectomy remains the cornerstone of management in this population when indicated, offering potential for substantial clinical improvement. Early detection through comprehensive diagnostic measures is paramount in preventing further morbidity. This case serves as a stark reminder of the destructive potential of dysregulated calcium-phosphorus metabolism in end-stage renal disease.
